# Platelet count and clinical outcomes in hospitalized patients with COVID-19 pneumonia

**DOI:** 10.3389/fmed.2025.1614447

**Published:** 2025-10-10

**Authors:** Carmine Siniscalchi, Pierpaolo Di Micco, Angela Guerra, Riccardo Simoni, Julia Magro, Alberto Parise, Nicoletta Cerundolo, Egidio Imbalzano, Claudio Tana, Lorenzo Finardi, Tiziana Meschi

**Affiliations:** ^1^Department of Internal Medicine, Parma University Hospital, Parma, Italy; ^2^AFO Medicina PO Santa Maria delle Grazie, Naples, Italy; ^3^Division of Internal Medicine, Department of Clinical and Experimental Medicine, University of Messina, Messina, Italy; ^4^Geriatrics Clinic, University Hospital of Chieti, Chieti, Italy

**Keywords:** COVID, platelet, venous thromboembolism, pneumonia, respiratory insufficiency

## Abstract

**Background:**

Thrombocytopenia has been associated with poor outcomes in various infectious diseases, including COVID-19. This study investigates the relationship between platelet (PLT) count at hospital admission and clinical characteristics, treatments, and outcomes in patients with COVID-19 pneumonia.

**Methods:**

We retrospectively analyzed 797 patients hospitalized for COVID-19 pneumonia, stratifying them into three groups by platelet count: <150,000/mm^3^ (22%), 150,000–400,000/mm^3^ (76%), and >400,000/mm^3^ (2.5%).

**Results:**

Patients with PLT < 150,000/mm^3^, more frequently male, and had a higher prevalence of cirrhosis and fibrosis. They presented less severe respiratory impairment and lower inflammatory markers. They also showed lower use of enoxaparin and a higher use of fondaparinux. Mortality was at the limits of significance in this group (37% vs. 28 and 20%, *p* = 0.056), and thrombocytopenia was independently associated with increased risk of in-hospital death (HR 1.483, 95% CI 1.023–2.150; *p* = 0.037).

**Conclusion:**

Thrombocytopenia on admission independently predicts mortality in patients hospitalized with COVID-19 pneumonia.

## Introduction

Coronavirus disease 2019 (COVID-19), caused by the severe acute respiratory syndrome coronavirus 2 (SARS-CoV-2), has resulted in a global public health emergency since its emergence in late 2019. Although the clinical spectrum of COVID-19 ranges from asymptomatic infection to severe respiratory failure and death, although the clinical spectrum of COVID-19 ranged from asymptomatic infection to severe respiratory failure and death, during the early phase of the pandemic a significant proportion of patients with pneumonia developed critical illness requiring hospitalization, oxygen supplementation, or ICU support. Understanding predictors of poor outcomes is therefore paramount in guiding clinical management, triage, and resource allocation.

Among the laboratory markers associated with adverse outcomes in COVID-19, platelet (PLT) count has received increasing attention. Platelets are small anucleate blood components that primarily mediate hemostasis and thrombosis, but they are also deeply involved in inflammation, immunity, and endothelial integrity (1–3). Beyond their role in hemostasis, platelets are now recognized as key players in innate immunity, interacting with leukocytes and endothelial cells to modulate the inflammatory response (1–3). In sepsis, thrombocytopenia is a well-known marker of severity and predicts poor outcomes (4, 5). In COVID-19, platelet count can be affected by bone marrow suppression, increased consumption due to microthrombi formation, immune-mediated destruction, or sequestration in inflamed lungs (6–9). Conversely, thrombocytosis may reflect cytokine-driven megakaryopoiesis and systemic hyperinflammation (10). These dynamic changes may help identify patients at higher risk for complications.” An increased platelet count may indicate prolonged inflammation and a heightened risk of thrombotic events (6), whereas thrombocytopenia may be associated with haemorrhagic complications (often worsened by concomitant antithrombotic therapy) or with thrombotic microangiopathies (TMA) and disseminated intravascular coagulation (DIC) (11). The role of thrombocytopenia in predicting poor prognosis in several infectious diseases has been already recognized, in particular in sepsis and community-acquired pneumonia (4, 5). In COVID-19, thrombocytopenia has been variably reported across cohorts, with some studies linking it to disease severity and mortality (6–9). Conversely, thrombocytosis has also been documented and may reflect systemic inflammation or underlying comorbid conditions (10).

Several pathophysiological mechanisms have been proposed to explain platelet count alterations in COVID-19. SARS-CoV-2 infection may lead to direct or immune-mediated megakaryocyte suppression, bone marrow infiltration, or increased peripheral destruction of platelets (7, 12, 13). Moreover, COVID-19 has been associated with disseminated intravascular coagulation (DIC), thrombotic microangiopathy, and a hyperinflammatory cytokine storm, all of which may contribute to platelet consumption and activation (11, 14, 15). Platelets may also act as amplifiers of inflammation through their interactions with leukocytes and endothelial cells, modulating cytokine release and vascular permeability (3, 16, 17). Despite the growing interest in the prognostic value of platelet count, the evidence remains inconsistent, particularly regarding its association with inflammatory response, thrombotic events, and the need for respiratory support. Furthermore, the prognostic significance of thrombocytopenia versus thrombocytosis in hospitalized COVID-19 patients is not fully clarified, and few studies have evaluated platelet count alongside other established prognostic markers such as gas exchange, creatinine, or inflammatory biomarkers (18, 19).

To address this gap, we conducted a retrospective analysis of a large cohort of patients hospitalized for COVID-19 pneumonia during the early phase of the pandemic. We aimed to investigate the association between admission platelet count and clinical characteristics, radiological severity, laboratory parameters, therapeutic approaches, and clinical outcomes including mortality and venous thromboembolism (VTE). We also explored the independent predictive value of platelet count when included in multivariable prognostic models, controlling for key confounders such as age, comorbidities, gas exchange, and systemic inflammation.

This study provides insights into the utility of platelet count as a readily available biomarker for risk stratification in COVID-19 pneumonia and contributes to a better understanding of its pathophysiological and clinical implications.

## Materials and methods

### Study design and population

This retrospective observational cohort study was conducted at Parma University Hospital in Italy which was appointed as the main hub for the care of SARS-CoV-2 patients for the whole Parma province (approximately 450,000 inhabitants) in the earliest phases of the first wave. We included consecutive adult patients (≥18 years old) admitted with a confirmed diagnosis of COVID-19 pneumonia. COVID-19 infection was confirmed by reverse transcription polymerase chain reaction (RT-PCR) on nasopharyngeal swabs. Pneumonia was diagnosed based on clinical symptoms and compatible imaging findings on chest CT (23). A total of 797 patients were enrolled. Patients were excluded if they lacked platelet count data at admission or had missing data for critical covariates. The cohort was stratified into three groups according to platelet count at admission: thrombocytopenia (<150,000/mm^3^), normal range (150,000–400,000/mm^3^), and thrombocytosis (>400,000/mm^3^), as defined in prior literature (7, 9). Demographic, clinical, and laboratory data were extracted from electronic medical records using a standardized template, similar to prior retrospective COVID-19 cohorts. The CT visual severity score was calculated as the estimated percentage of lung involvement, as previously validated. Treatment data included antiviral agents, hydroxychloroquine (off-label use), corticosteroids, and antithrombotic therapies such as enoxaparin and fondaparinux (18, 19). Primary outcomes were in-hospital mortality and venous thromboembolism (VTE), as confirmed by imaging modalities following standard diagnostic protocols (14). Other outcomes included bleeding, non-invasive ventilation (NIV), ICU admission, and length of stay. Ethics Committee approval was obtained (Comitato Etico dell’Area Vasta Emilia Nord, Emilia-Romagna region) under the ID 273/2020/OSS/AOUPR as part of a larger project on the characteristics of patients hospitalized with confirmed or suspected COVID-19 during the first pandemic wave. All participants, who were contactable by phone or for follow-up reasons, provided written informed consent for participation. For all other cases, the Ethics Committee, in accordance with the guidelines in force at the moment of approval, waived written informed-consent collection due to the retrospective design of the study.

### Statistical analysis

Continuous variables were reported as median with interquartile range (IQR) and compared using the Kruskal–Wallis test. Categorical variables were expressed as frequencies and percentages and compared using the Chi-square test. Trends across platelet groups were assessed using the Jonckheere–Terpstra test for continuous variables and Mantel–Haenszel test for categorical variables. To evaluate the independent effect of platelet count on in-hospital mortality and VTE, we performed a Cox proportional hazards regression for time-to-event analysis (in-hospital mortality), and a Logistic regression analysis for binary outcomes (mortality, VTE). Models were adjusted for clinically relevant variables and potential confounders selected based on prior knowledge and univariate analysis. A stepwise forward selection method was applied, retaining variables with *p* < 0.05. Hazard ratios (HR) and odds ratios (OR) with 95% confidence intervals (CI) were reported. Statistical significance was set at a two-sided *p*-value < 0.05. Multivariable Cox and logistic regression models identified independent predictors of mortality and VTE, following guidelines for observational COVID-19 studies (30, 31). Analyses were performed with the SPSS statistical package (v. 29, IMB, Armonk, NY, USA), considering *p* values < 0.05 as statistically significant.

The CHA_2_DS_2_-VASc score was included as a global measure of comorbidity burden and vascular risk, not solely for its original indication in atrial fibrillation. In the logistic regression analysis, we included variables recorded at patient admission that showed a significant *p*-value after stratification of the population for VTE. The application of the stepwise method allowed the selection of independent predictors among these variables.

## Results

**Figure 1:**
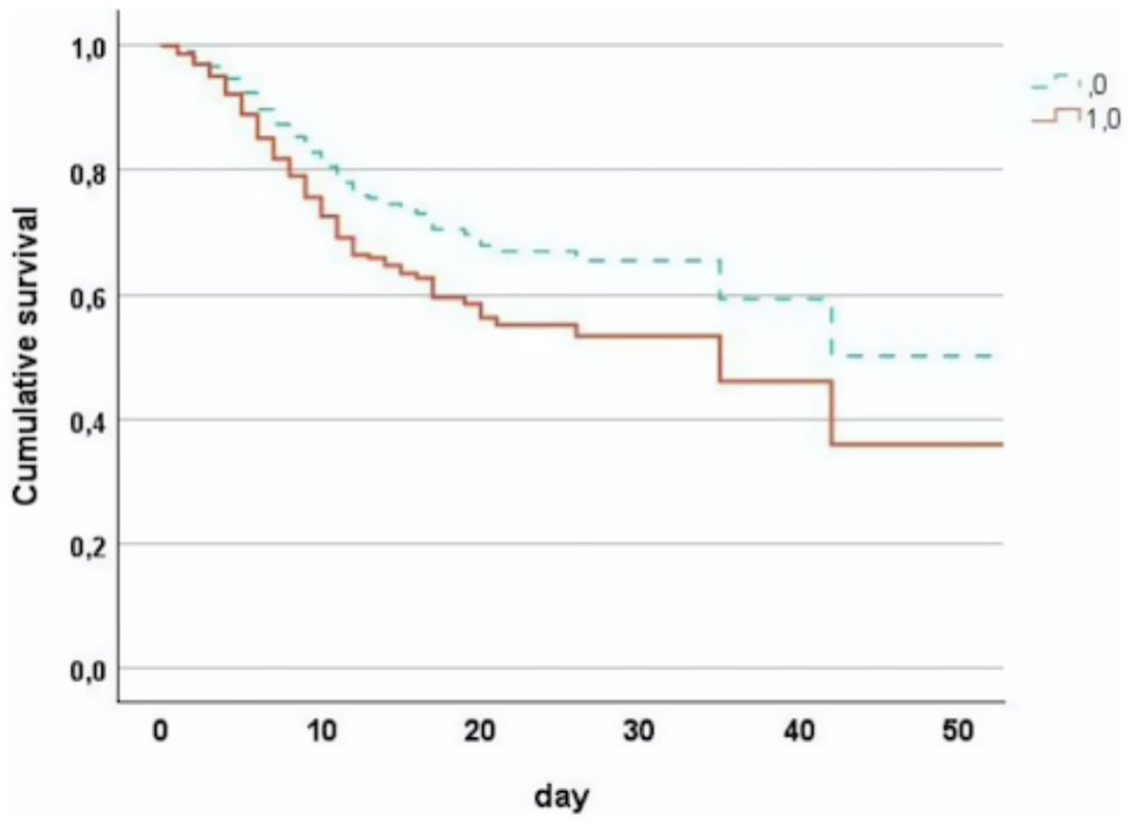
Cumulative survival in first-wave covid pneumonia patients stratified by PLT < 150,000/mm^3^ (1) and PLT ≥ 150,000/mm^3^ (0).

A total of 797 patients hospitalized with confirmed COVID-19 pneumonia were included in the analysis. The median age was 74 years (IQR 62–82), and 42% were female. The distribution of patients by platelet (PLT) count was as follows: 174 patients (21.8%) with thrombocytopenia (<150,000/mm^3^), 603 patients (75.5%) with normal platelet count (150,000–400,000/mm^3^), and 20 patients (2.5%) with thrombocytosis (>400,000/mm^3^) (Table 1). Patients with thrombocytopenia tended to be older (median age 76 vs 73 vs 73 years), although this difference was not statistically significant (*p* = 0.390). Female sex was significantly more common in the thrombocytosis group (70%) compared to the thrombocytopenia (37%) and normal PLT groups (43%) (*p* = 0.015; *p* for trend = 0.021). Regarding comorbidities, the overall burden was comparable among groups. However, cirrhosis and pulmonary fibrosis were significantly more prevalent in the thrombocytopenic group (cirrhosis: 5% vs 1% vs 0%, *p* = 0.011; fibrosis: 3% vs 1% vs 0%, *p* = 0.028). No significant differences were observed in rates of hypertension, diabetes, heart disease, cancer or COPD (Figure 1).

**Table 1:** Anamnestic characteristics of patients hospitalized for COVID-19 pneumonia stratified by platelets. (PLT): <150,000/mm^3^, 150,000–400,000/mm^3^ and >400,000/mm^3^.

N.797	PLT <150,000/mm^3^ N.174 (22%)	PLT 150,000–400,000/mm^3^ N.603 (76%)	PLT >400,000/mm^3^ N.20 (2.5%)	*p*	*P* for trend
Age, years	76 (64–83)	73 (62–82)	73 (60–83)	0.390	0.208
Female gender, %	37	43	70	**0.015**	**0.021**
Comorbidities					
Chronic comorbidities, number	3 (2–5)	3 (1–4)	3 (1–6)	0.182	0.145
CHA_2_DS_2_-Vasc score	3 (1–4)	3 (1–4)	4 (1–5)	0.248	0.773
Hypertension, %	57	60	50	0.515	0.727
Diabetes, %	19	21	26	0.718	0.469
Heart disease, %	28	24	30	0.535	0.477
Obesity, %	11	12	0	0.252	0.622
Cancer, %	18	14	5	0.221	0.104
IRC, %	9	6	0	0.290	0.157
COPD, %	11	11	5	0.667	0.692
Dementia, %	14	12	20	0.519	0.834
Cerebral vasculopathy, %	7	8	15	0.474	0.565
Stroke outcomes, %	5	5	15	0.184	0.362
Cirrhosis, %	5	1	0	**0.011**	**0.003**
Osteoporosis, %	6	7	20	0.055	0.177
Fibrosis, %	3	1	0	**0.028**	**0.009**
Chronic drugs					
Systemic drugs, number	4 (1–6)	3 (1–6)	5 (1–8)	0.648	0.969
ACE inhibitors, %	24	25	37	0.461	0.455
Sartani, %	17	16	11	0.778	0.650
Calcium Antagonists, %	16	23	11	0.064	0.164
Beta-blockers, %	38	32	47	0.105	0.326
Vasodilators, %	2	3	0	0.651	0.772
Insulin, %	5	6	21	**0.019**	0.072
Diuretics, %	30	28	53	0.073	0.595
Statins/lipid lowering drugs, %	24	26	32	0.740	0.504
Corticosteroids, %	6	7	11	0.722	0.511
Fans, %	1	1	0	0.893	0.743
Anti-platelet agents, %	27	33	37	0.382	0.166
TAO/NAO, %	16	11	21	0.105	0.248
Antidepressants, %	15	16	21	0.729	0.480
Alpha-lytics, %	9	10	0	0.336	0.750
Antiepileptics, %	4	6	11	0.396	0.191
Antipsychotics, %	5	8	11	0.492	0.235

Data reported as median and IQR or percentage. *p* calculated with Kruskal–Wallis or chi square, *p* for trend calculated with Jonckheere Terpstra or Mantel Haenszel. *P* values < 0.05 are indicated in bold.

Symptoms on admission, including fever, cough, and diarrhea, were similar across groups (Table 2). Dyspnea was more frequent in thrombocytosis patients (70%) than in normal PLT (54%) or thrombocytopenia groups (39%) (*p* < 0.001). CT visual severity score showed a progressive increase from thrombocytopenia to thrombocytosis (25% vs. 30% vs. 50%, *p* < 0.001), indicating greater pulmonary involvement in patients with elevated PLT. Table 2 summarizes laboratory findings. Thrombocytopenic patients showed significantly better oxygenation (pO_2_/FiO_2_ ratio: 296 vs 231 vs 200; *p* < 0.001), higher creatinine (1.0 vs. 0.9 vs. 0.8 mg/dL; *p* = 0.008), and lower lymphocyte counts (0.80 vs. 0.90 vs. 1.09 × 10^3^/mm^3^; *p* = 0.006). Neutrophil and monocyte counts increased progressively across PLT groups, suggesting an enhanced inflammatory response. C-reactive protein (CRP) and fibrinogen levels were significantly elevated in patients with normal or high PLT compared to thrombocytopenic patients (CRP: 68 vs. 108 vs. 116 mg/L, *p* < 0.001; fibrinogen: 502 vs. 629 vs. 666 mg/dL, *p* < 0.001), supporting a potential reactive thrombocytosis in severe inflammation.

**Table 2:** Clinical presentation and blood tests detected at admission in patients with COVID-19 pneumonia stratified by platelets (PLT): <150,000/mm^3^, 150,000–400,000/mm^3^ and >400,000/mm^3^.

N.797	PLT <150,000/mm^3^ N.174 (22%)	PLT 150,000–400,000/mm^3^ N.603 (76%)	PLT >400,000/mm^3^ N.20 (2.5%)	*p*	*P* for trend
Clinical presentation of COVID-19 on admission					
Symptom duration, days	7 (3–8)	7 (4–10)	7 (5–10)	0.519	0.308
Cough, %	45	49	50	0.648	0.368
Dyspnea, %	39	54	70	**<0.001**	**<0.001**
Fever, %	89	88	85	0.831	0.555
Diarrhea, %	7	7	0	0.480	0.506
Asthenia, %	11	9	25	0.071	0.712
Other symptoms, %	19	15	15	0.335	0.170
CT visual score, %	25 (15–40)	30 (20–50)	50 (38–61)	**<0.001**	**<0.001**
Positive swab, %	100	100	100		
Arterial blood gas analysis on admission					
pH	7.45 (7.42–7.47)	7.45 (7.42–7.48)	7.45 (7.43–7.52)	0.548	0.340
HCO_3_^−^, mmol/L	25 (22–27)	25 (23–27)	25 (23–28)	0.336	0.154
pCO_2_, mmHg	36 (33–39)	36 (32–39)	38 (34–41)	0.238	0.268
pO_2_, mmHg	74 (62–94)	73 (61–90)	80 (57–109)	0.492	0.346
pO_2_/FiO_2_	296 (164–376)	231 (120–324)	200 (91–231)	**<0.001**	**<0.001**
Clinical chemistry and hematology on admission					
Hemoglobin, g/dL	13.8 (12.0–14.9)	13.6 (12.3–14.7)	13.3 (11.0–14.8)	0.500	0.330
Neutrophilis, 1,000/mm^3^	3.31 (2.21–5.10)	5.42 (3.77–7.61)	8.10 (4.56–10.92)	**<0.001**	**<0.001**
Lymphocytes, 1,000/mm^3^	0.80 (0.55–1.11)	0.90 (0.63–1.21)	1.09 (0.87–1.66)	**0.006**	**0.003**
Monocytes, 1,000/mm^3^	0.33 (0.22–0.47)	0.40 (0.27–0.56)	0.61 (0.31–0.75)	**<0.001**	**<0.001**
Creatinine, mg/dL	1.0 (0.8–1.3)	0.9 (0.7–1.1)	0.8 (0.6–1.1)	**0.008**	**0.002**
Sodium, mEq/L	137 (135–139)	138 (135–140)	138 (134–139)	0.221	0.130
Potassium, mEq/L	4.0 (3.7–4.3)	4.0 (3.7–4.3)	4.1 (3.6–4.7)	0.575	0.464
Creatine-phosphokinase, IU/L	160 (85–339)	136 (72–328)	82 (59–164)	0.114	0.162
Lactate-dehydrogenase, IU/L	329 (263–425)	356 (270–485)	395 (306–498)	**0.040**	**0.012**
Aspartate aminotransferase, IU/L	44 (33–70)	46 (32–79)	38 (29–61)	0.522	0.603
D-Dimer, ng/mL	931 (605–2,169)	972 (629–1,620)	862 (634–6,399)	0.876	0.861
INR ratio	1.20 (1.12–1.29)	1.21 (1.13–1.32)	1.18 (1.12–1.32)	0.594	0.322
aPTT ratio	1.00 (0.90–1.08)	0.98 (0.90–1.06)	1.04 (0.94–1.11)	0.233	0.460
Fibrinogen, mg/dL	502 (395–612)	629 (513–754)	666 (596–898)	**<0.001**	**<0.001**
C-reactive protein, mg/L	68 (33–127)	108 (55–175)	116 (42–161)	**<0.001**	**<0.001**
Procalcitonin, ng/mL	0.17 (0.09–0.50)	0.17 (0.08–0.48)	0.12 (0.06–0.27)	0.290	0.309

Data reported as median and IQR or percentage. *p* calculated with Kruskal–Wallis or chi square, *p* for trend calculated with Jonckheere Terpstra or Mantel Haenszel. *P* values < 0.05 are indicated in bold.

Antithrombotic therapy varied significantly across groups. Enoxaparin was less frequently administered in thrombocytopenic patients (77%) than in those with normal (94%) or high PLT (100%) (*p* < 0.001), whereas fondaparinux was used more often in the thrombocytopenia group (23% vs. 5% vs. 0%, *p* < 0.001). Hydroxychloroquine off-label use was higher in patients with thrombocytosis (85%) (*p* = 0.019). No significant differences were found in the use of antibiotics, steroids, or other anti-inflammatory drugs (Table 3).

**Table 3:** Clinical course and outcomes in patients hospitalized for COVID-19 pneumonia stratified by platelets (PLT): <150,000/mm^3^, 150,000–400,000/mm^3^ and >400,000/mm^3^.

N.797	PLT <150,000/mm^3^ N.174 (22%)	PLT 150,000–400,000/mm^3^ N.603 (76%)	PLT >400,000/mm^3^ N.20 (2.5%)	*p*	*P* for trend
Therapies against COVID-19					
Antiviral drugs, %	61	65	65	0.589	0.336
Antibiotics, %	98	96	100	0.443	0.614
Linezolid, %	9	6	15	0.135	0.288
Anti-inflammatories	74	79	90	0.202	0.098
Hydroxychloroquine (off-label), %	63	73	85	**0.019**	**0.005**
Steroids, %	21	20	30	0.568	0.810
Fans, %	6	4	0	0.225	0.088
Enoxaparin, %	77	94	100	**<0.001**	**<0.001**
Dose enoxaparin, UI	6,000 (4,000–8,000)	6,000 (4,000–8,000)	6,000 (4,000–6,000)	0.242	0.550
Fondaparinux, %	23	5	0	**<0.001**	**<0.001**
Dose fondaparinux, mg	2.5 (1.5–5.0)	2.5 (1.5–7.5)	/	0.417	0.417
Outcomes					
VTE, %	0	3	5	0.078	**0.024**
Bleeding, %	3	3	5	0.852	0.855
NIV, %	8	12	5	0.186	0.287
ICU, %	3	5	0	0.280	0.470
Death, %	37	28	20	0.056	**0.017**
Length of stay, day	7 (3–12)	7 (4–12)	8 (4–12)	0.514	0.252

Data reported as median and IQR or percentage. *p* calculated with Kruskal–Wallis or chi square, *p* for trend calculated with Jonckheere Terpstra or Mantel Haenszel. *P* values < 0.05 are indicated in bold.

### Clinical outcomes

In-hospital mortality was highest among thrombocytopenic patients (37%), followed by those with normal PLT (28%) and thrombocytosis (20%). Although the overall *p* value was marginal (*p* = 0.056), the trend was statistically significant (*p* for trend = 0.017) (Table 3).

VTE occurred in 0% of thrombocytopenic, 3% of normal PLT, and 5% of thrombocytosis patients (*p* = 0.078; *p* for trend = 0.024). Bleeding rates were low and not significantly different across groups. NIV was required in 8% of thrombocytopenic, 12% of normal PLT, and 5% of thrombocytosis patients (*p* = 0.186). ICU admission occurred in 3, 5, and 0% of patients, respectively. Median length of stay was similar between groups: 7 days in thrombocytopenia and normal PLT groups, and 8 days in the thrombocytosis group (*p* = 0.514). (Table 3).

### Multivariable analysis

Cox regression analysis (method stepwise) revealed that age (HR 1.063, 95% CI 1.046–1.080; *p* < 0.001), CT score (HR 1.030, 95% CI 1.021–1.039; *p* = 0.005), pO_2_/FiO_2_ (HR 0.995, 95% CI 0.993–0.997; *p* < 0.001), creatinine (HR 1.202, 95% CI 1.046–1.380; *p* = 0.009), and CRP (HR 1.003, 95% CI 1.001–1.006; *p* = 0.006) were independently associated with risk of death. Importantly, PLT < 150,000/mm^3^ was independently associated with risk of variables associated with mortality.

Logistic regression identified PLT group (OR 4.447, 95% CI 1.238–15.967; *p* = 0.022), hemoglobin (OR 1.410, 95% CI 1.097–1.812; *p* = 0.007), and CHA_2_DS_2_-VASc score (OR 1.386, 95% CI 1.052–1.826; *p* = 0.020) as independent predictors of thromboembolic events (Table 4).

**Table 4:** Risk of death in hospital in patients with COVID-19 pneumonia tested with cox regression multivariate analysis stepwise method.

	*p*	Hazard ratio	95% CI for hazard ratio
Age, years	<0.001	1.063	1.046–1.080
Chest CT visual score, %	0.005	1.030	1.021–1.039
pO_2_/FiO_2_	<0.001	0.995	0.993–0.997
Creatinine, mg/dL	0.009	1.202	1.046–1.380
C-reactive protein, mg/L	0.006	1.003	1.001–1.006
PLT < 150,000/mm^3^ vs. PLT ≥ 150,000/mm^3^	0.037	1.483	1.023–2.150

Covariates: age, sex, pathologies number, creatininemia, cirrhosis, fibrosis, granulocytes, lymphocytes, monocytes, CT visual score, pO_2_/FiO_2_, fibrinogen, Creactive protein, LDH and PLT.

PLT was entered both as a continuous variable, in dichotomous form: <100,000/mm^3^ and ≥100,000/mm^3^, <150,000/mm^3^ and ≥150,000/mm^3^ and stratified into three groups: <150,000/mm^3^, 150,000–400,000/mm^3^ and >400,000/mm^3^.

We stratified thrombocytopenic patients into three groups: mild (100–150 × 10^3^/μL), moderate (50–99 × 10^3^/μL), and severe (<50 × 10^3^/μL). Mortality in the severe thrombocytopenia group was 67%, although this subgroup included only three patients, limiting the statistical significance of this finding when compared with the other two groups. Mortality in patients with moderate thrombocytopenia was 52% (*n* = 42) and in those with mild thrombocytopenia 31% (*n* = 129), *p* = 0.031. Mortality was significantly higher in the moderate group compared with the mild group (OR 2.420, 95% CI 1.188–4.930, *p* = 0.015). In contrast, mortality in patients with mild thrombocytopenia (31%) was not significantly higher than in those with a platelet count ≥150 × 10^3^/μL (28%; OR 1.164, 95% CI 0.770–1.758, *p* = 0.472) (Table 5).

**Table 5:** Factors independently associated with mortality in patients hospitalized for COVID-19 pneumonia tested with logistic regression model multivariate analysis, stepwise method.

	*p*	Odds ratio	95% CI for odds ratio
Age, years	<0.001	1.080	1.056–1.105
Chest CT visual score, %	0.004	1.017	1.006–1.029
pO_2_/FiO_2_	<0.001	0.993	0.990–0.995
Creatinine, mg/dL	0.003	1.363	1.110–1.674
PLT, 1,000 mm^3^	0.008	0.996	0.993–0.999

Covariates: age, sex, pathologies number, creatininemia, cirrhosis, fibrosis, granulocytes, lymphocytes, monocytes, CT visual score, pO_2_/FiO_2_, fibrinogen, Creactive protein, LDH and PLT.

(PLT was entered both as a continuous variable, in dichotomous form: <150,000/mm^3^ and ≥150,000/mm^3^ and stratified into three groups: <150,000/mm^3^, 150,000–400,000/mm^3^ and >400,000/mm^3^).

Among patients with platelet counts <150,000/mm^3^, only nine did not receive anticoagulation; in this subgroup, mortality was 33% compared with 37% in those who received anticoagulation (*p* = 0.431) (Table 6).

**Table 6:** Factors independently associated with VTE in patients hospitalized for COVID-19 pneumonia tested with logistic regression model multivariate analysis, stepwise method.

	*p*	Odds ratio	95% CI for odds ratio
PLT (Groups, uncategorized)	0.022	4.447	1.238–15.967
Hemoglobin, g/dL	0.007	1.410	1.097–1.812
CHA_2_DS_2_-Vasc score	0.020	1.386	1.052–1.826

## Discussion

In this large retrospective cohort of patients hospitalized with COVID-19 pneumonia, we found that platelet count at admission was significantly associated with clinical severity, laboratory abnormalities, and in-hospital outcomes, including mortality and venous thromboembolism (VTE). Notably, thrombocytopenia (<150,000/mm^3^) emerged as an independent predictor of mortality after adjustment for age, oxygenation parameters, renal function, and inflammation markers. Conversely, higher platelet counts were associated with increased risk of VTE, suggesting a dual prognostic implication of platelet dynamics in COVID-19. While thrombocytopenia was more frequent in patients with cirrhosis or fibrosis, subgroup analysis showed that thrombocytopenia remained an independent predictor of death in multivariable analysis even after adjusting for the presence of liver disease. These findings suggest that low platelet count is not solely a surrogate marker for liver dysfunction.

The association between thrombocytopenia and adverse outcomes has been reported across multiple infectious and critical illnesses, such as sepsis, community-acquired pneumonia, and dengue fever or in several viral infection in which the association with TMA has been frequently reported. (1, 2, 10) In COVID-19, early reports from Wuhan indicated a higher prevalence of thrombocytopenia in critically ill patients (3). Our findings indicate that patients with normal platelet counts or thrombocytosis exhibited more severe respiratory impairment, as evidenced by a higher prevalence of dyspnoea, greater radiological involvement on chest CT, and lower pO_2_/FiO_2_ ratios. Multiple mechanisms may contribute to thrombocytopenia in SARS-CoV-2 infection. Bone marrow suppression due to viral infiltration or cytokine-mediated damage, increased platelet consumption secondary to immune-mediated destruction or thrombotic microangiopathy, and sequestration in inflamed pulmonary vasculature have all been postulated (6–8). Platelets themselves may contribute to the immune dysregulation seen in COVID-19, as they can release cytokines, form aggregates with leukocytes, and activate the endothelium (12, 13). Thus, a low platelet count may reflect the burden of underlying comorbidities such as liver disease or immune dysregulation, rather than being an active contributor to disease pathogenesis.

Our analysis showed that thrombocytopenia was independently associated with in-hospital mortality, even after adjusting for known predictors such as age, gas exchange, CT severity score, creatinine, and CRP. The hazard ratio (HR 1.483) aligns with previously published estimates ranging from 1.5 to 3.5 depending on the severity of thrombocytopenia and the population studied (6, 11). Importantly, the independent prognostic value of PLT count underscores the potential role of routine hematological parameters in early risk stratification. Interestingly, thrombocytosis (>400,000/mm^3^), although rare (2.5% of patients), was associated with higher inflammatory markers and VTE incidence, but not with increased mortality. This may reflect a reactive thrombocytosis driven by systemic inflammation, rather than a specific pathogenic mechanism per se. The absence of excess mortality in this group might also be due to its small size, limiting statistical power. COVID-19 is associated with a prothrombotic state characterized by elevated D-dimer, endothelial dysfunction, and cytokine-induced hypercoagulability, leading to both arterial and venous thrombotic events (14, 15). Our study confirms the association between elevated platelet count and risk of VTE. Multivariable logistic regression identified PLT group as an independent predictor of thromboembolic events, alongside hemoglobin levels, and CHA_2_DS_2_-VASc score. These findings align with the concept that platelet activation may play a role in COVID-associated coagulopathy (16). Interestingly, thrombocytopenic patients had no observed VTE events, but this might be partly explained by a more cautious anticoagulation approach or underdiagnosis due to clinical severity. Moreover, fondaparinux use was higher in this group, potentially reflecting concerns over heparin-induced thrombocytopenia (HIT) or clinician preference for synthetic agents in thrombocytopenic settings. While observational, this finding suggests the need to tailor anticoagulation strategies based on platelet count and clinical context.

Anticoagulation remains a cornerstone of COVID-19 management, particularly in hospitalized VTE patients with moderate to severe disease. Recent guidelines recommend prophylactic or therapeutic doses of low molecular weight heparin (LMWH) depending on disease severity, D-dimer levels, VTE and bleeding risk. (17, 18). In our study, thrombocytopenic patients were less likely to receive enoxaparin and more likely to be treated with fondaparinux, perhaps due to perceived safety. However, whether this shift in anticoagulation modality impacts outcomes requires further prospective evaluation. Furthermore, the low overall rate of major bleeding across groups supports the safety of anticoagulation, even in patients with moderate thrombocytopenia (>50,000/mm^3^). These findings align with emerging evidence suggesting that thrombocytopenia alone should not contraindicate anticoagulation unless the count is critically low.

Our results highlight the importance of routine platelet count as a simple and cost-effective prognostic marker in COVID-19. Thrombocytopenia should prompt clinicians to consider early escalation of care. Elevated platelet count, on the other hand, may signal heightened inflammatory and thrombotic risk, warranting intensified monitoring and possibly therapeutic anticoagulation. Future guidelines could consider incorporating platelet thresholds in risk stratification algorithms for hospitalized patients. From a pathophysiological perspective, the dual role of platelets as markers of severity and mediators of vascular inflammation warrants further study. Platelet activation markers, immature platelet fraction, and platelet-leukocyte aggregates may provide more refined prognostic tools beyond simple count measurements.

Several limitations of this study merit consideration. First, the retrospective nature limits causal inference. Second, platelet count was assessed only at admission; dynamic changes during hospitalization may provide additional prognostic value. Third, the relatively small size of the thrombocytosis group may have underpowered some comparisons. Fourth, although adjusted analyses included major confounders, residual confounding cannot be excluded.

Despite limitations, this study has several strengths. It includes a large, well-characterized cohort of hospitalized COVID-19 pneumonia patients, with detailed laboratory, clinical, and outcome data. Platelet count was evaluated both categorically and continuously, and rigorous multivariable models were used to assess independent associations. The inclusion of both mortality and VTE as outcomes allows a comprehensive appraisal of the prognostic implications of platelet count.

## Conclusion

This study highlights platelet count as a powerful and easily accessible biomarker in COVID-19. Thrombocytopenia at admission independently predicts mortality, reinforcing previous studies. Thrombocytosis, though rare, is associated with VTE and elevated inflammatory markers, suggesting the need for closer monitoring and possibly intensified antithrombotic prophylaxis. Given the ease and cost-effectiveness of platelet count measurement, this parameter should be incorporated into early risk stratification strategies for COVID-19. Moreover, therapeutic decisions, particularly regarding anticoagulation, may benefit from platelet-guided approaches to balance thrombotic and bleeding risks. These results support the integration of platelet count into early risk stratification tools and treatment algorithms for COVID-19 pneumonia. Future studies should explore dynamic changes in platelet parameters and assess the efficacy of platelet-guided anticoagulation strategies.

## Data Availability

The raw data supporting the conclusions of this article will be made available by the authors, without undue reservation.
